# Discovery of autophagy as a universal mechanism for sex steroid synthesis in human ovary and testis

**DOI:** 10.1080/27694127.2023.2251804

**Published:** 2023-08-30

**Authors:** Yashar Esmaeilian, Francesko Hela, Gamze Bildik, Ece İltumur, Sevgi Yusufoglu, Kayhan Yakin, Ozgur Oktem

**Affiliations:** a Research Center for Translational Medicine, Koç University, 34450, Turkiye; b The Graduate School of Health Sciences, Koç University, Istanbul, 34450, Turkiye; c Department of Experimental Therapeutics, the University of Texas MD Anderson Cancer Center, Houston, TX 77030, USA; d Department of Obstetrics and Gynecology, Koç University School of Medicine, Istanbul, Turkiye

**Keywords:** Autophagy, sex steroids, steroidogenesis, ovary, testis, human, gonadotropins, luteal phase defect

## Abstract

We recently discovered that lipophagy is a key mechanism to provide free cholesterol required for steroid biosynthesis in human ovary and testis. Pharmacological or genetic inhibition of autophagy by silencing of the autophagy-related (*ATG*) genes *BECN1* (*BECLIN1*) and *ATG5* resulted in a significant reduction in basal and gonadotropin-stimulated estradiol, progesterone (P_4_) and testosterone production in the ex-vivo explant tissue and cell culture models for ovary and testis. We also described a new mechanism of action for gonadotropin hormones, i.e., follicle stimulating hormone (FSH) and human chorionic gonadotropin (hCG)/luteinizing hormone (LH), in this process. They augment the production of sex steroid hormones by upregulating the expression of *ATG* genes, the accelerating autophagic flux and promoting LDs sequestration into autophagosomes and degradation in lysosomes. Furthermore, we detected several molecular aberrations at different steps of lipophagy-dependent P_4_ production in the ovary of women with defective luteal function. Our findings might have important clinical implications by opening a new avenue for the understanding and treatment of a wide range of diseases varying from reproductive disorders to sex hormone-producing neoplasms and hormone dependent malignancies, such as carcinomas of breast, endometrium, and prostate.

**Abbreviations:** ACAT, Acyl-coenzyme A-cholesterol-acyl-transferase; AMBRA1, autophagy and beclin 1 regulator 1; ATG, autophagy-related; BECN1, BECLIN1; hCG, human chorionic gonadotropin; E_2,_ estradiol; FSH, follicle stimulating hormone; GABARAP, GABA type A receptor-associated protein; GCs, luteinized granulosa cells; IVF, *in vitro* fertilization; LAMP2A, lysosomal associated membrane protein 2A; LDs, lipid droplets; LDLs, low-density lipoproteins; P_4_: progesterone; PCOS, polycystic ovary syndrome; SOAT1: Sterol-O-acetyltransferase; MAP1LC3B, microtubule associated protein 1 light chain 3 beta; PLIN3, perilipin 3; STAR, steroidogenic acute regulatory protein; SQSTM1, sequestosome-1.

Steroid hormones, including sex steroids [androgens, estrogens, and progesterone (P_4_)] and the others (glucocorticoid and mineralocorticoid hormones), are synthesized from cholesterol in a complex biosynthetic pathway. The main source of steroidogenic cholesterol are the circulating low-density lipoproteins (LDLs), which are internalized by receptor-mediated endocytosis after binding to their receptors. In late compartments of the endosomal system, lysosomal acid lipases cleave the ester group of cholesterol esters bound to the NPC intracellular cholesterol transporter 2 (NPC2) and the resulting unesterified cholesterol is then transferred to NPC intracellular cholesterol transporter 1, a membrane-associated cholesterol binding protein, which is inserting into the limiting membrane of these organelles and controls cholesterol efflux from lysosomes. Free cholesterol released from lysosomes is then either transported to mitochondria by cytosolic and mitochondrial proteins or stored in the lipid droplets (LDs) following re-esterification by SOAT1 (sterol O-acetyltransferase 1). In mitochondria, cholesterol is transported form the outer mitochondrial membrane to inner membrane mainly via a carrier protein called STAR (steroidogenic acute regulatory protein) for steroid hormone production. Cytoplasmic LDs are the major source of cholesterol as up to 80% of the total cholesterol is found esterified in these organelles in steroidogenic cells. The intracellular trafficking of cholesterol for steroid hormone synthesis has been the subject of extensive research for the last 50 years, but it has still not been fully elucidated. We have very recently revealed that lipophagy, a selective form of autophagy, plays a pivotal role in sex steroid hormone biosynthesis by mediating lysosomal degradation of LDs to release free cholesterol for steroid hormone biosynthesis in human ovary and testis [1].

Human primary luteinized granulosa cells (GCs), obtained from either *in vitro* fertilization (IVF) patients during an oocyte retrieval procedure or corpus luteum tissue samples obtained from the patients undergoing ovarian surgery, were used to study the role of lipophagy in the P_4_ biosynthesis branch of steroidogenesis. In contrast, testicular tissue samples and non-luteinized granulosa cells were used to investigate testosterone (T) and estrogen (estradiol, E_2_) biosynthesis, respectively. We observed that luteotropic hormone human chorionic gonadotropin (hCG) not only up-regulates the expression of STAR and enhances P_4_ production of the luteinized GCs, but it also decreases the generation of lipidated LC3B (microtubule-associated protein 1 light chain 3 beta), which is an autophagy substrate that is degraded following the fusion of the autophagosome with lysosome. In this regard, pharmacological inhibition of the autophagy with chloroquine, which blocks lysosomal hydrolysis stage or vinblastine, which impairs autophagosome-lysosome fusion, resulted in a significant drop in P_4_, E_2_ and T production, which was accompanied with an accumulation of lapidated LC3B and sequestosome-1 (SQSTM1), an autophagosome cargo proteins, and a marked cytoplasmic accumulation of lipids mainly as LDs. Confocal microscopy also revealed that LDs associate with lysosomes to a certain extent as evidenced by co-localization of PLIN3 (perilipin 3), a LDs-associated protein, with LAMP2 (lysosome-associated membrane protein-2), a lysosome marker protein, and this co-localization was increased after hCG and particularly after this hormone was added with the lysosomal inhibitor chloroquine. Interestingly, such an increase in PLIN3/LAMP2 co-localization was not observed in luteinized GCs when autophagy was pharmacologically inhibited with vinblastine or genetically blocked by silencing the *ATG5* gene. Basal and hCG-stimulated P_4_ production significantly decreased in these cells in which autophagy was inhibited, indicating the importance of autophagosome formation in the delivery of LDs into lysosomes. In support of these findings, confocal imaging after triple staining for LC3B, PLIN3 and LAMP2 revealed that PLIN3 not only co-localizes with lysosome but also with LC3B and these co-localizations markedly increased after gonadotropin treatment of GCs. By contrast, labeling of free cholesterol with filipin showed no colocalization between LC3B and free cholesterol in confocal images, indicating that autophagosome specifically deliver LDs into lysosomes.

Perhaps more notably, we have detected several defects in lipophagy-mediated P_4_ steroidogenesis in the luteinized GCs from patients known to have a dysfunctional corpus luteum, which is a transient endocrine organ that produces P_4_ hormone, and it is required for both pregnancy establishment and maintenance. These patients displayed a reduction in the expression of several *ATG* genes [i.e., *autophagy and beclin 1 regulator 1* (*AMBRA1*), *ATG16L1, ATG4D, ATG5, BECN1, GABA type A receptor-associated protein (GABARAP*), *GABARAPL1* and *GABARAPL2*] assessed by both qRT-PCR and western blot, incomplete degradation of lapidated LC3B-II and defective delivery of LDs into lysosomes.

In conclusion our work revealed that lipophagy is a key mechanism in basal and gonadotropin-stimulated sex steroid synthesis in human ovary and testis, and it provides new insights into the molecular pathogenetic mechanism of defective P_4_ production in the GCs from patients with compromised luteal function. Our schematic model of how lipophagy contributes to sex steroid biosynthesis in human gonads is shown in the Figure 1. Sex steroid hormones control diverse physiological processes from sexual differentiation, somatic growth, metabolic function, reproduction, pregnancy and immunity to gender-specific differences in brain function and behavior. They also have a role in the development of specific cancers such as the ones of breast, endometrium and prostate, autoimmune disease like systemic lupus erythematosus (SLE), endocrine disorders including polycystic ovary syndrome (PCOS) and sex steroid producing neoplasms. Therefore, investigation of lipophagy-mediated sex steroidogenesis in those diseases and neoplasms may help to understand better their pathogenesis at the molecular level and develop new alternative therapeutic strategies.

**Figure 1.**
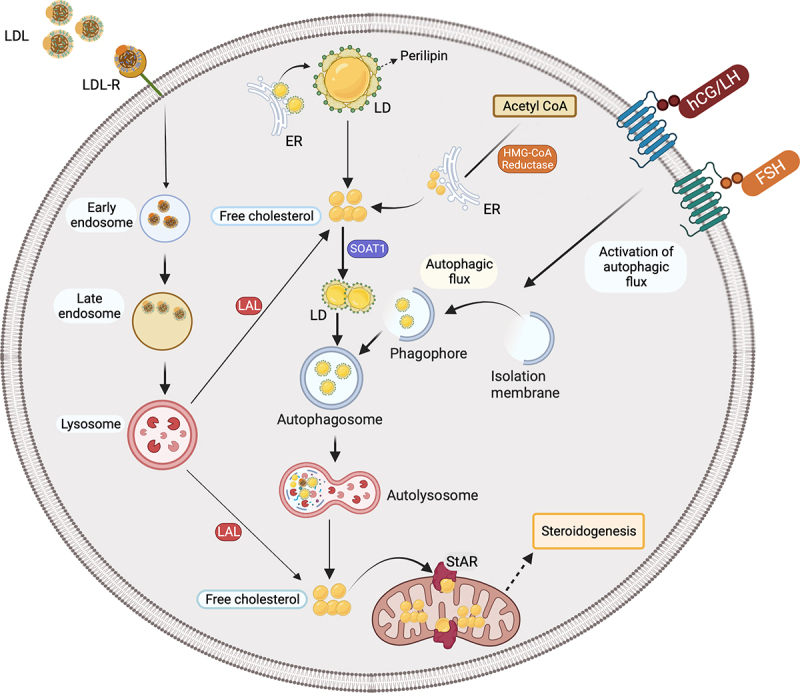
Schematic diagram of the contribution of lipophagy to sex steroid hormone biosynthesis in human gonads. Cholesterol for steroidogenesis is acquired mainly from plasma lipoproteins or de novo synthesis from acetyl coenzyme A. Lipoprotein-gathering receptors of the low-density lipoprotein (LDL) receptor family located on microvilli projected from plasma membrane mediate lipoprotein uptake by an endocytic mechanism that delivers them to the lysosomes, where the apolipoproteins are degraded. The resulting cholesterol esters are then hydrolyzed by acid lipases to release free cholesterol. Free cholesterol is either transported to mitochondria for steroidogenesis or stored in LDs after re-esterification by SOAT1. Autophagy mediates the association of the cholesterol-loden LDs with lysosome to deliver their lipid cargo into lysosomes for degradation (lipophagy), to release the free cholesterol required for steroid synthesis. Gonadotropin hormones, i.e., FSH and hCG/LH, augment sex steroid production by accelerating autophagic flux and promoting the fusion of the LDs with lysosome to generate more free cholesterol available for steroidogenesis.
